# An Incidental Cavitary Pulmonary Lesion Unveiling Diffuse Large B-cell Lymphoma in an Undiagnosed HIV Patient

**DOI:** 10.7759/cureus.93326

**Published:** 2025-09-27

**Authors:** Mansi Jain, Dhayananth Rattaipalivalasu Saravanan, Vijay K Doddapaneni, Manogna Pendyala, Vinod Khatri

**Affiliations:** 1 Internal Medicine, St. Vincent's Medical Center, Toledo, USA; 2 Department of Internal Medicine, St. Vincent's Medical Center, Toledo, USA; 3 Pulmonary and Critical Care Medicine, St. Vincent's Medical Center, Toledo, USA

**Keywords:** cavitary lung lesion, diffuse large b cell lymphoma (dlbcl), hiv, hiv lymphoma, incidental

## Abstract

Diffuse large B-cell lymphoma (DLBCL) rarely presents with pulmonary involvement, and cavitary lung lesions as the initial manifestation are exceptionally uncommon. We report the case of a 65-year-old man who presented to the emergency department after a mechanical fall, during which imaging revealed an incidental thin-walled cavitary lesion in the left lung apex. The patient was asymptomatic and was lost to follow-up. Six months later, he presented again with a new right axillary mass. Imaging demonstrated a persistent cavitary lesion with bilateral axillary lymphadenopathy and a new solid lung nodule in the left lower lobe. Biopsies confirmed DLBCL in both the lung and lymph node. He was also diagnosed with a previously unrecognized HIV infection, with a CD4 count of 84 cells/mm³. The patient was started on antiretroviral therapy and R-CHOP chemotherapy. This case highlights the importance of maintaining a broad differential diagnosis for cavitary lung lesions, pursuing tissue diagnosis even in asymptomatic patients, and ensuring robust follow-up of incidental findings to avoid delays in diagnosis and treatment.

## Introduction

Diffuse large B cell lymphoma (DLBCL) is the most common subtype of non-Hodgkin lymphoma (NHL), accounting for approximately 30% of NHL cases in patients without HIV infection and up to 45% of cases in those with HIV related lymphoma [1]. DLBCL usually arises in lymph nodes but may also involve sites outside the lymphatic system (extranodal disease), such as the gastrointestinal tract, central nervous system, and, less commonly, the lungs [2]. Pulmonary involvement at the time of initial diagnosis is rare and usually presents with respiratory complaints (such as cough or shortness of breath) or systemic "B symptoms" including fever, weight loss, and night sweats. On imaging, pulmonary DLBCL often appears as mass-like opacities or areas of consolidation. Cavitary lung lesions, which are air-filled spaces within abnormal lung tissue, are particularly uncommon in DLBCL and are generally linked to symptomatic disease [3-14]. Because DLBCL is an aggressive lymphoma, symptoms typically develop within weeks to a few months of disease onset. An interval of up to six months between the first radiographic abnormality and the subsequent detection of lymphadenopathy, as in our case, is unusual and highlights the potential for silent progression. Given the broad differential for cavitary lung lesions, including infections, vasculitides, and malignancies, obtaining a tissue diagnosis is essential, especially when lesions are detected incidentally and the patient has no symptoms [15]. HIV associated DLBCL is particularly aggressive and often presents with advanced disease, making early recognition especially important in this population.

We report a case of incidentally detected cavitary pulmonary DLBCL in a patient who was subsequently diagnosed with HIV. This case underscores the importance of diagnostic vigilance and reliable follow-up systems to prevent incidental radiographic findings from being overlooked.

## Case presentation

**Figure 1:**
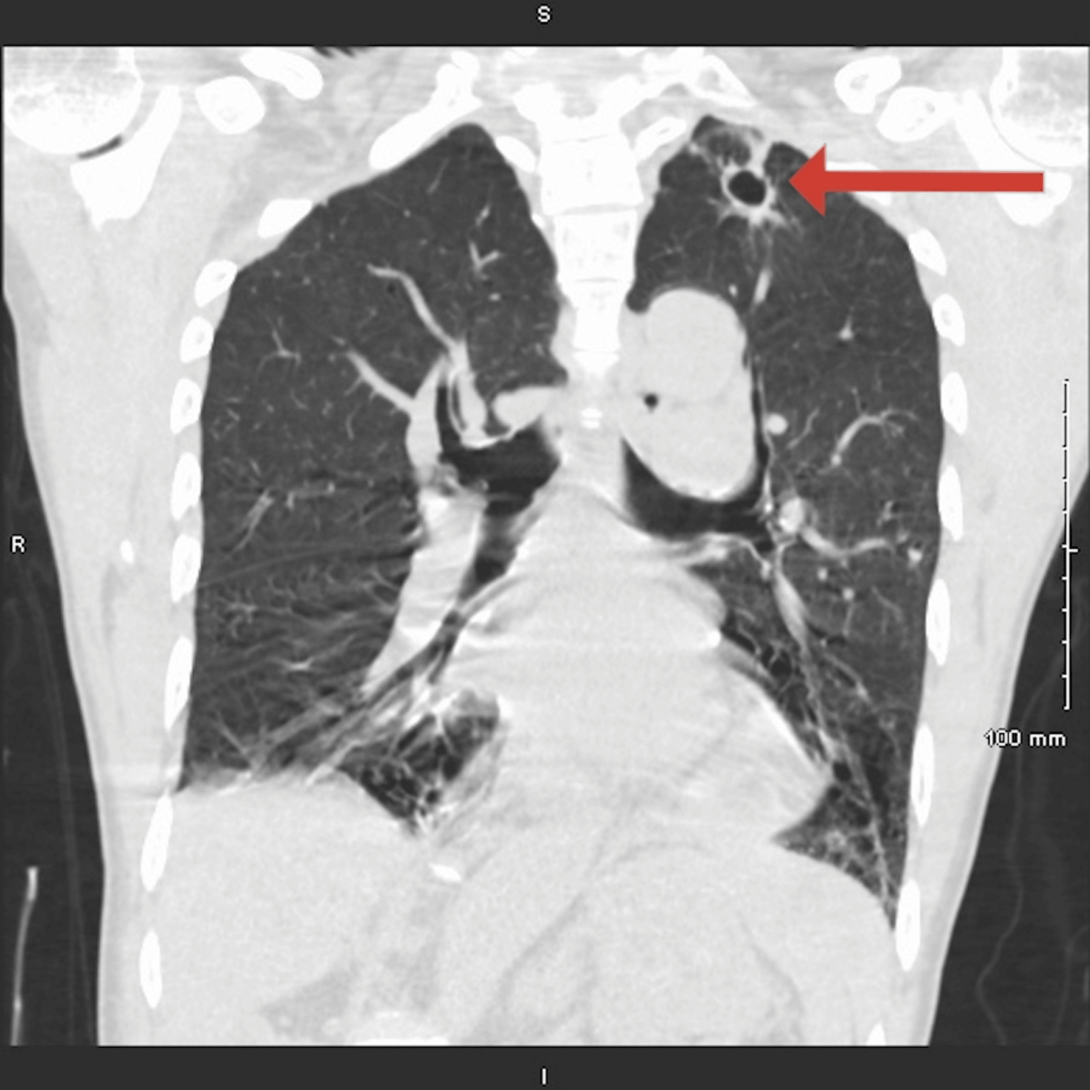
Non-contrast CT image of the chest demonstrating a cavitary lesion in the apex of the left lung, as indicated by the red arrow

A 65-year-old male with no significant past medical history initially presented to the emergency department (ED) after sustaining a mechanical fall. He reported neck pain, and a CT cervical spine revealed no acute fractures, though multilevel degenerative disc disease and facet arthropathy were identified throughout the cervical spine. Incidentally, a cavitary lesion was identified in the apex of the left lung. A non-contrast CT scan of the chest confirmed the presence of a thin-walled cavitary lesion without surrounding consolidation or lymphadenopathy (Figure 1).

The patient denied respiratory symptoms, including cough, dyspnea, hemoptysis, fever, night sweats, or weight loss. He had no history of recent travel, tuberculosis exposure, or occupational exposure to asbestos, silica, or other known carcinogens. He worked in a kitchen environment. He was discharged with symptomatic treatment (cyclobenzaprine) for the neck pain and advised to follow up with a pulmonologist and obtain a high-resolution CT chest (HRCT), which was not completed.

Six months later, the patient re-presented to the ED with a new, painless mass in the right axilla, which he had noticed one week earlier. It was non-tender, mobile, and without overlying erythema or fluctuance. He remained afebrile without systemic symptoms. A bedside ultrasound of the mass showed no cystic component or signs of cellulitis. Laboratory results from that visit are shown in Table 1.

**Table 1:** Complete blood count

Parameter	Result	Reference range (male)
White blood cell count (WBC) (×10³/µL)	2.5	3.5–11.3 ×10³/µL
Red blood cell count (RBC) (×10⁶/µL)	3.76	4.2–5.9 ×10⁶/µL
Hemoglobin concentration (g/dL)	10.7	13.5–17.5 g/dL
Hematocrit (%)	33.0	41–53%
Mean corpuscular volume (MCV) (femtoliters)	87.8	80–100 fL
Mean corpuscular hemoglobin (MCH) (picograms)	28.5	27–33 pg
Mean corpuscular hemoglobin concentration (MCHC) (g/dL)	32.4	32–36 g/dL
Mean platelet volume (MPV) (femtoliters)	9.2	7.5–11.5 fL
Red cell distribution width (RDW) (%)	14.2	11.5–14.5%
Platelet count (×10³/µL)	152	150–450 ×10³/µL
Neutrophil percentage (%)	41	40–70%
Lymphocyte percentage (%)	40	20–45%
Monocyte percentage (%)	14	2–10%
Eosinophil percentage (%)	4	1–6%
Basophil percentage (%)	1	0–2%
Absolute neutrophil count (×10³/µL)	1.03	1.5–8.0 ×10³/µL
Absolute lymphocyte count (×10³/µL)	1.00	1.0–4.0 ×10³/µL
Absolute monocyte count (×10³/µL)	0.36	0.2–0.8 ×10³/µL
Absolute eosinophil count (×10³/µL)	0.11	0.02–0.5 ×10³/µL
Absolute basophil count (×10³/µL)	<0.03	0.0–0.2 ×10³/µL
Immature granulocyte percentage (%)	0	0–0.4%
Absolute immature granulocyte count (×10³/µL)	<0.03	0.0–0.3 ×10³/µL
Nucleated red blood cells (NRBC), automated count (per 100 WBCs)	0.0	0

**Figure 2:**
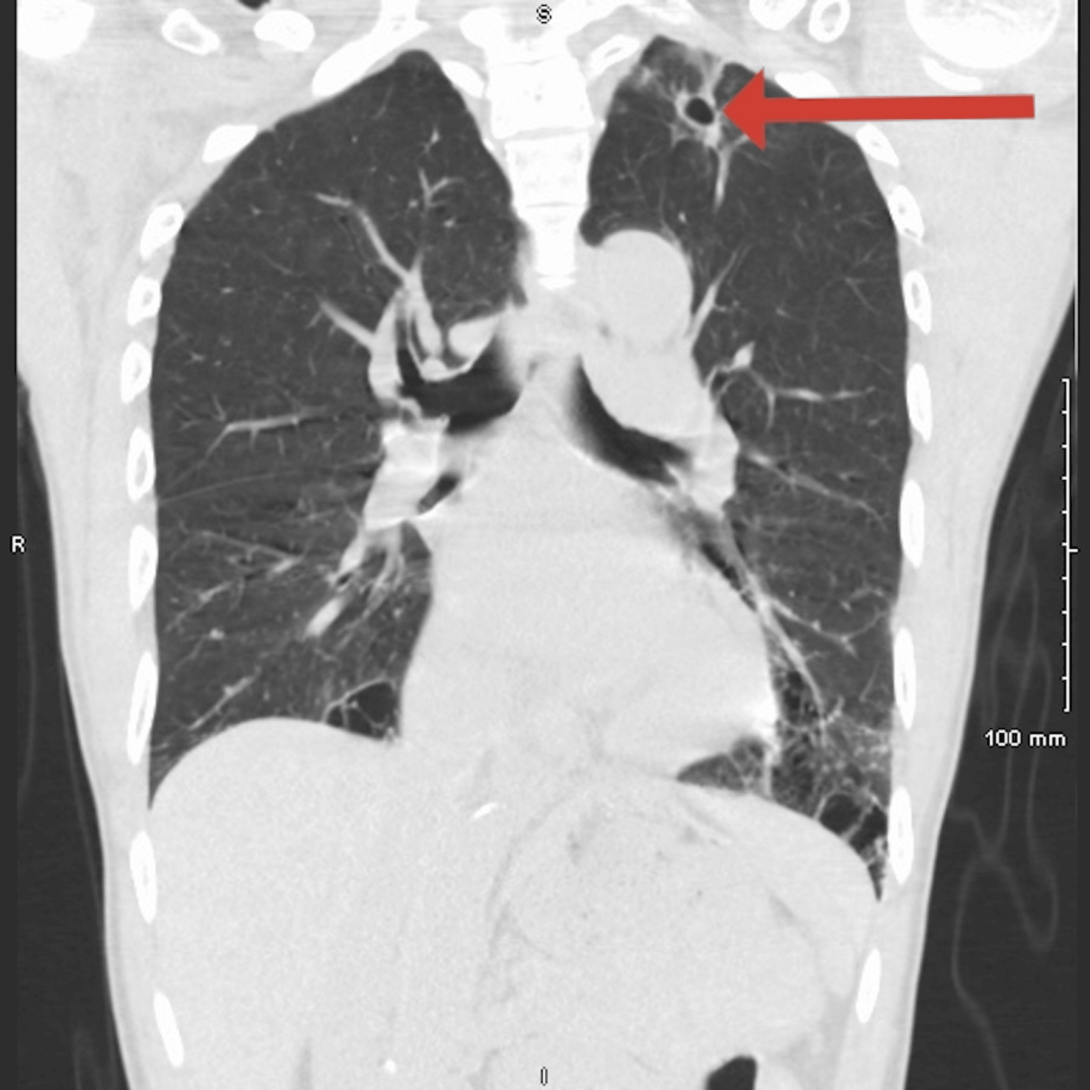
Non-contrast CT image of the chest demonstrating a slightly decreased left apical cavitary lesion, as indicated by the red arrow

**Figure 3:**
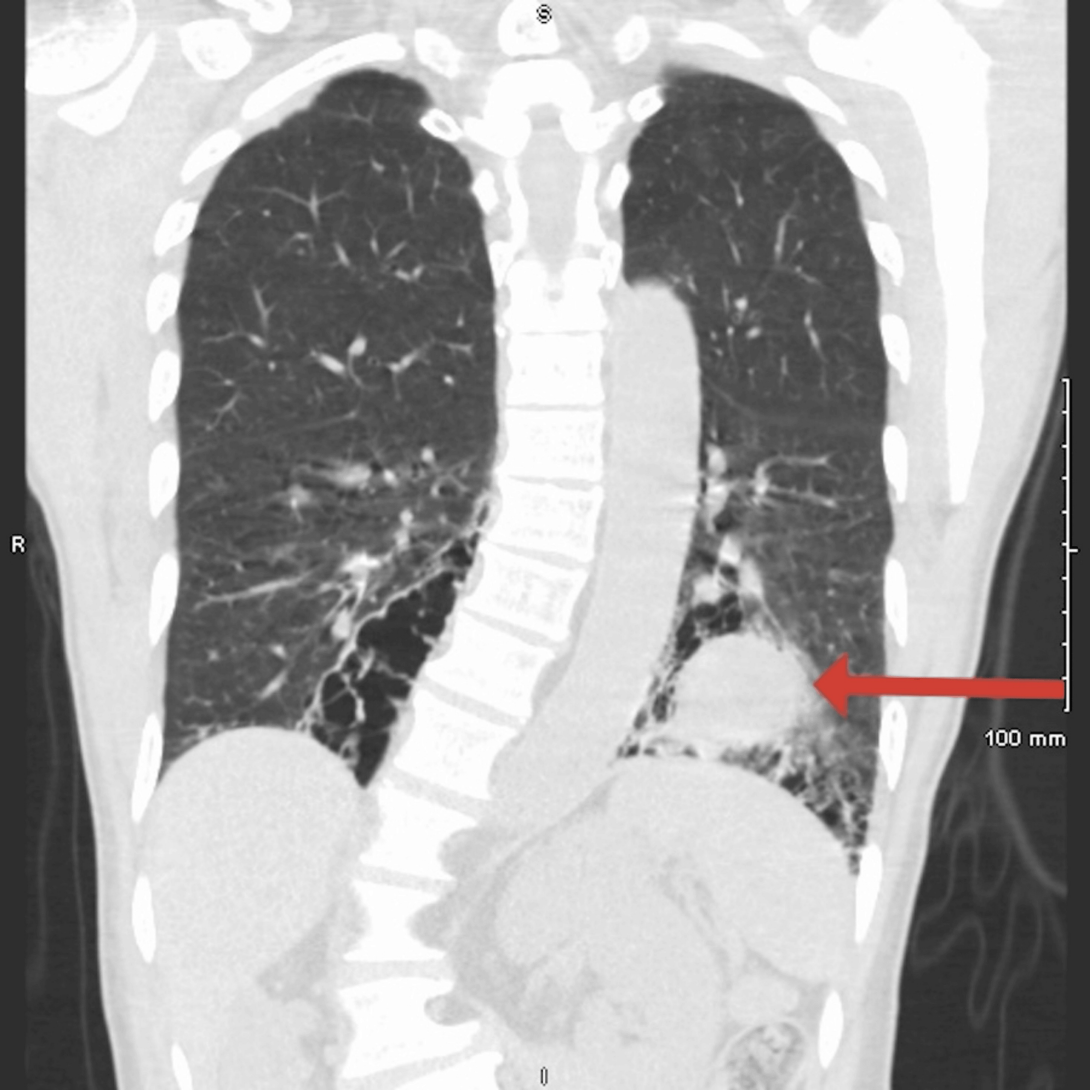
Non-contrast CT image of the chest demonstrating a solid nodule in the left lower lobe, as indicated by the red arrow

He was advised to follow up with his primary care physician (PCP) and pulmonologist. A repeat non-contrast chest CT showed a slightly decreased left apical cavitary lesion (Figure 2), but a new 4 cm solid nodule was noted in the left lower lobe (LLL) (Figure 3).

**Figure 4:**
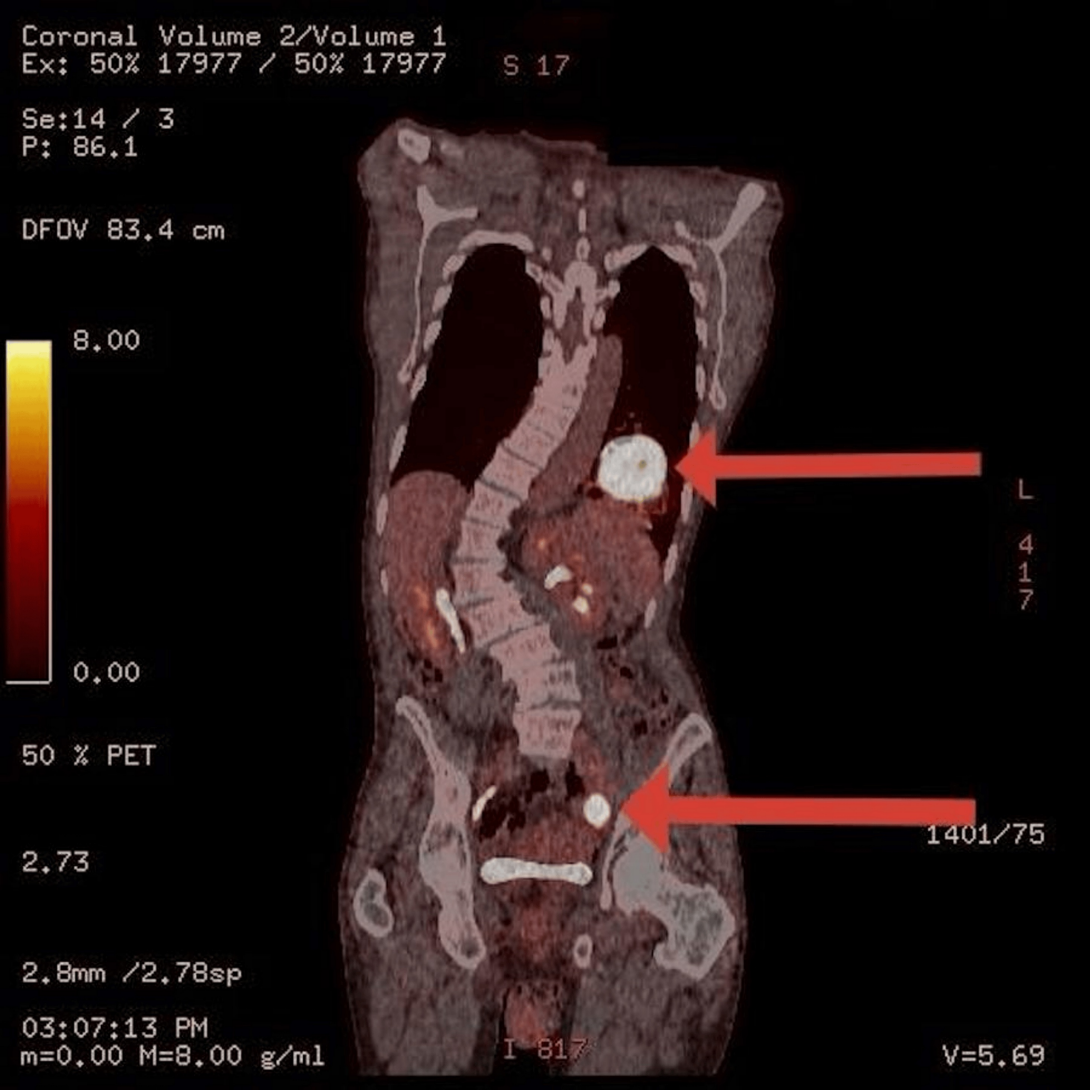
A PET-CT scan image demonstrating a metabolically active necrotic left lower lobe mass and left internal iliac lymph node, as indicated by the red arrows PET - positron emission tomography

**Figure 5:**
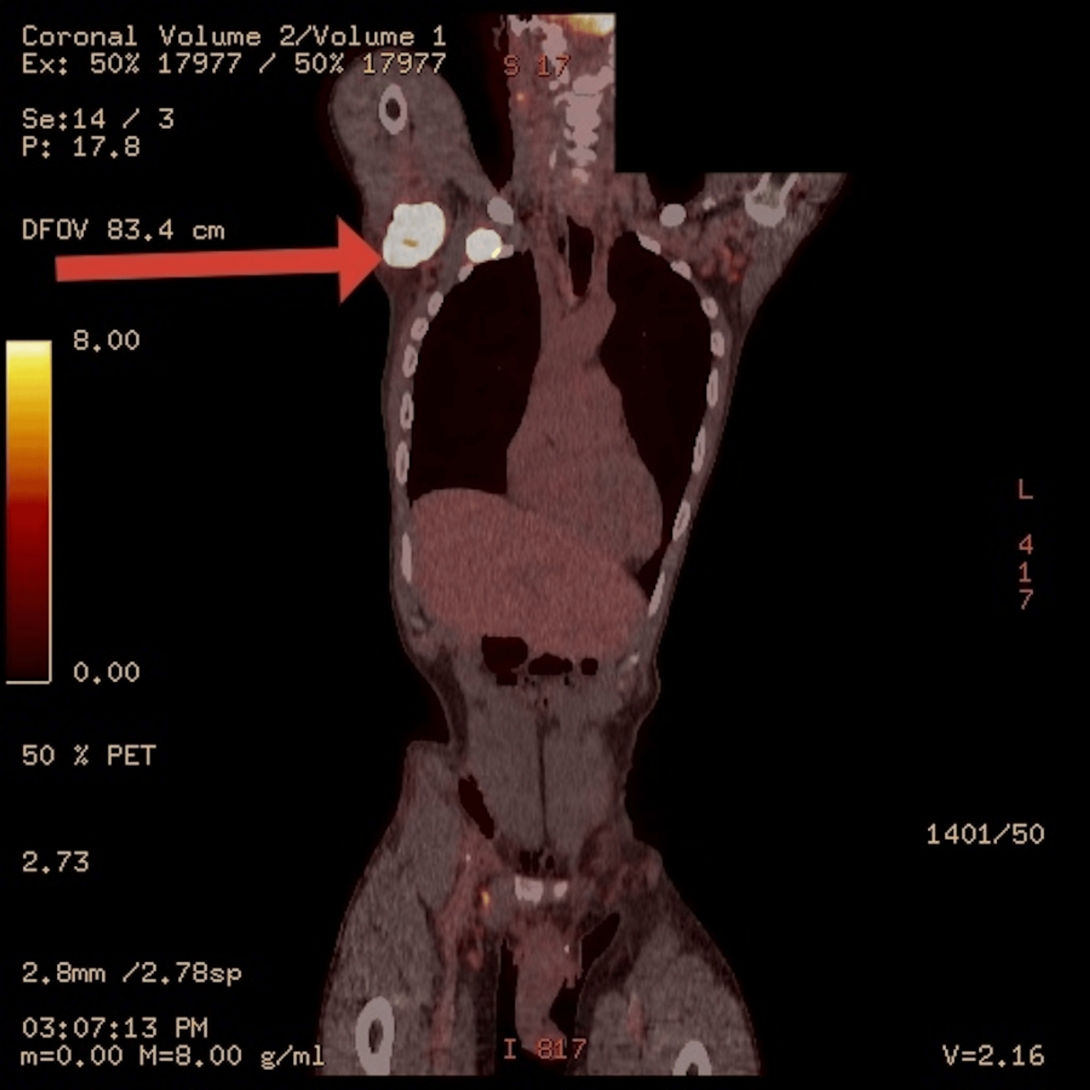
A PET-CT scan image demonstrating a metabolically active right axillary lymph node, as indicated by the red arrow PET - positron emission tomography

A positron emission tomography (PET)-CT scan demonstrated a metabolically active necrotic LLL mass, with hypermetabolic lymphadenopathy in the right and left axillae and possibly the left internal iliac and cervical chains, suggestive of malignancy (Figures 4, 5). A core biopsy of the right axillary lymph node revealed diffuse large B-cell lymphoma (DLBCL). Bronchoscopy and endobronchial biopsy of the lung lesion confirmed DLBCL. Acid-fast bacilli staining was negative.

Around this time, the patient presented with grayish penile discharge. He denied fever, dysuria, urinary frequency, urgency, or other systemic symptoms. Nucleic acid amplification testing (NAAT) was positive for *Chlamydia trachomatis* and *Neisseria gonorrhoeae*. He was treated with intramuscular ceftriaxone and a short course of doxycycline.

Given the concurrent diagnosis of DLBCL and a recent sexually transmitted infection, HIV testing was performed and returned positive. His absolute CD4 T-cell count was 84 cells/mm³ (reference: 309-1571 cells/mm³) with an HIV RNA load of 54,000 copies/mL. The VDRL test was negative, and the Quantiferon-TB Gold test yielded an indeterminate result. G6PD activity was mildly reduced (9 U/g Hb; reference: 9.9-16.6 U/g Hb).

The patient was referred to Infectious Diseases and initiated on antiretroviral therapy (bictegravir/emtricitabine/tenofovir alafenamide) and atovaquone for Pneumocystis jirovecii pneumonia prophylaxis.

He subsequently followed up with the Hematology-Oncology department. MRI of the brain showed no evidence of CNS involvement, and lumbar puncture was negative for malignant cells. He received intrathecal methotrexate prophylactically and was started on R-CHOP chemotherapy (rituximab, cyclophosphamide, doxorubicin, vincristine, and prednisone).

This case illustrates an unusual presentation of diffuse large B-cell lymphoma (DLBCL) with pulmonary involvement in a newly diagnosed HIV-positive patient.

## Discussion

Pulmonary involvement in NHL is an uncommon but important manifestation, occurring in approximately 4% of patients at the time of initial diagnosis [2]. DLBCL in HIV-positive individuals tends to be more aggressive with a poorer prognosis than in immunocompetent patients [16]. Typically, pulmonary DLBCL presents with respiratory symptoms, systemic B symptoms such as fever, night sweats, or weight loss, and radiologic findings of mass lesions, consolidations, or infiltrates [2]. Cavitary lesions are rare and are hypothesized to result from a high disease burden, causing central necrosis [3,4]. In our review of 12 case reports where DLBCL was associated with a cavitary lung lesion, all patients were symptomatic, either with respiratory symptoms or B symptoms, unlike our case, where the disease remained clinically silent [3-14]. This contributed to pulmonary lymphoma being considered lower on the differential diagnosis for a cavitary lesion, contrasting sharply with prior reports where cavitary DLBCL lung lesions presented with overt symptoms, highlighting the importance of diagnostic flexibility in the workup of cavitary lesions.

The differential diagnosis for cavitary lung lesions is broad and often leads to initial consideration of infectious, inflammatory, or vascular etiologies. In regions with a high prevalence of tuberculosis (TB), this remains a leading concern, particularly in immunosuppressed individuals. However, in such settings, especially among HIV positive patients, the clinical and radiologic overlap between infectious and malignant etiologies poses a significant diagnostic challenge. This underscores the importance of maintaining a broad differential diagnosis, even when TB seems likely, and helps contextualize the rarity of lymphoma as a cause of cavitary lesions. Other common considerations include bacterial lung abscesses, fungal infections (such as aspergillosis), septic emboli, vasculitides (such as granulomatosis with polyangiitis), and primary or metastatic malignancies [15]. The rarity of lymphoma as a cause of cavitary lesions, especially in asymptomatic patients, underscores the need for tissue diagnosis in unexplained cases to avoid misdiagnosis and delayed treatment.

This case also highlights the critical role of maintaining a closed diagnostic loop for incidental findings. However, the cavitary lesion was recognized initially; follow-up imaging and specialist consultation were not completed, contributing to a delayed diagnosis. A structured system to track incidental findings and ensure appropriate follow-up could have facilitated earlier tissue diagnosis and management. Ensuring that incidental pulmonary lesions are not lost to follow-up is essential to prevent missed or delayed diagnoses of serious conditions.

## Conclusions

This case highlights a rare presentation of diffuse large B-cell lymphoma (DLBCL) as an incidental cavitary lung lesion in an asymptomatic patient, ultimately leading to the diagnosis of advanced HIV. Unlike previously reported symptomatic cases, the silent nature of this presentation underscores the importance of obtaining tissue diagnosis for unexplained pulmonary lesions and ensuring timely follow-up of incidental findings. It serves as a reminder that even incidental and asymptomatic abnormalities can conceal serious disease, and that vigilance in these settings can make a critical difference in patient outcomes.
